# P058: How accurate are our estimates of Staphylococcus aureus antibiotic resistance in Australia?

**DOI:** 10.1186/2047-2994-2-S1-P58

**Published:** 2013-06-20

**Authors:** S Azim, G Nimmo, M-L Mclaws

**Affiliations:** 1 SPHCM, UNSW Medicine, The University of New South Wales, Sydney, Australia; 2 Pathology Queensland, Queensland Health, Herston, Australia; 3 SPHCM, UNSW Medicine, The University of New South Wales, Sydney, Australia

## Introduction

The Australian Group on Antimicrobial Resistance (AGAR) provide national reports on Methicillin resistant and methicillin sensitive *Staphylococcus aureus* (MRSA) antibiogram patterns for 14 antibiotics based on a decade of using the First 100 clinical isolates from inpatients and outpatients tested in participating laboratories. The First 100 isolates provided by 5 Queensland hospitals to AGAR represent inpatient isolates for every second year, 2005 to 2009, and outpatients for every second year, 2000 to 2008. Validity of the resistance patterns idenitfied by the samples is imperative for national surveillance.

## Objectives

AGAR data were tested for ability to demonstrate resistance patterns similar to the years for full datasets.

## Methods

A percentage point (PP) difference between the antibiograms for the first 100 samples and the corresponding 12-month dataset was estimated as a measure of validity. Robustness of the consecutive sampling of the first 100 isolates was tested using 15 random iterations of 100 isolates from the corresponding full 12-month datasets for inpatients and outpatients. Validity and the effect of phenotypes on the AGAR resistant patterns was tested against 6 antibiotics for inpatients and 5 antibiotics for outpatients.

## Results

AGAR inpatient data for 2007 and 2009 demonstrated significantly higher resistance levels compared with the full dataset with exception of Clindamycin and Gentamicin for 2009. In the most recent outpatient sampling, 2008, AGAR estimated a significantly lower level of resistance to all 5 antibiotics. Resistance patterns identified from 15 iterations indicated random sampling does not improve the validity of in- or outpatient antibiograms. The resistance patterns for inpatient and outpatient full datasets were driven by AUS-2/3 and EMRSA15 phenotypes with 95% of resistance removed on removal of these phenotypes.

## Conclusion

Given the small numbers of MRSA isolates and the effect of endemic phenotypes valid antibiograms and annual fluctuations require the entire annual dataset for validity.

## Disclosure of interest

None declared

